# State estimation of a physical system with unknown governing equations

**DOI:** 10.1038/s41586-023-06574-8

**Published:** 2023-10-11

**Authors:** Kevin Course, Prasanth B. Nair

**Affiliations:** Institute for Aerospace Studies, University of Toronto, Toronto, Ontario Canada

**Keywords:** Mathematics and computing, Engineering

## Abstract

State estimation is concerned with reconciling noisy observations of a physical system with the mathematical model believed to predict its behaviour for the purpose of inferring unmeasurable states and denoising measurable ones^1,2^. Traditional state-estimation techniques rely on strong assumptions about the form of uncertainty in mathematical models, typically that it manifests as an additive stochastic perturbation or is parametric in nature^3^. Here we present a reparametrization trick for stochastic variational inference with Markov Gaussian processes that enables an approximate Bayesian approach for state estimation in which the equations governing how the system evolves over time are partially or completely unknown. In contrast to classical state-estimation techniques, our method learns the missing terms in the mathematical model and a state estimate simultaneously from an approximate Bayesian perspective. This development enables the application of state-estimation methods to problems that have so far proved to be beyond reach. Finally, although we focus on state estimation, the advancements to stochastic variational inference made here are applicable to a broader class of problems in machine learning.

## Abstract (web summary)

A parametrization strategy for stochastic variational inference with Markov Gaussian processes is presented for state estimation of a physical system whose underlying dynamical equations are partially or completely unknown.

## Main

State estimation, or data assimilation as it is often referred to in the geosciences, forms the backbone of modern machinery for fusing noisy sensor data with mathematical models of complex systems in many important areas of science, engineering and finance. For example, in robotics systems, state estimation underpins control and route-planning algorithms, as well as being useful in high-level decision-making^4,5^. In finance, state estimation has been applied to estimate stochastic volatility models^6^. In medical imaging, state estimation has been advanced to improve cardiac-imaging technologies^7^. In meteorology, state estimation is used to reconstruct weather in the past and make predictions about weather in the future^8,9^.

The field of state estimation can arguably trace its roots back to the work of the early nineteenth-century astronomers. In 1801, the then 24-year-old Gauss devised what could be called the first state-estimation algorithm. Using only Kepler’s laws along with ordinary least squares, Gauss accurately computed the orbit of the asteroid Ceres from limited, noisy observations^1,10^. Our more modern conception of the field originates in the foundational works of Kalman and Bucy in the early 1960s. The researchers at the Research Institute for Advanced Studies, building on the work of Wiener and Kolmogorov in the 1940s, published their now well-known filtering algorithms for linear systems in 1960 and 1961^11,12^. As a testament to the success of their work, it was the Kalman filter—extended by Schmidt’s group at the NASA’s Ames Research Center to nonlinear systems—that solved the Apollo guidance and navigation problem^13^. Guided by a revolution in computing power and in the types of sensor available, such as the Global Positioning System, laser imaging and digital cameras^1^, modern developments in state estimation have focused on nonlinear systems^14^, high-dimensional systems^15,16^ and deriving more accurate approximations^5^.

An essential prerequisite for state estimation is a mathematical model describing how the system evolves over time. It is well accepted that some degree of uncertainty is inevitable in the mathematical characterization of any real-world system. Standard state-estimation algorithms assume that uncertainty in the governing equations manifests as an additive stochastic perturbation^1^ or is parametric in nature^3,17–20^. In reality, the true nature of uncertainty in mathematical models is much more diverse.

A more complete picture of uncertainty in mathematical models shows that it often also enters as a by-product of modelling errors^21^. Modelling errors are pernicious because they often arise owing to imperfect knowledge of the underlying system in combination with the need to simplify for computational considerations. For example, meteorology often uses empirical assumptions to approximate sub-gridscale processes^22^. Such modelling errors can be expressed mathematically as uncertainty in the structural form of the equations governing how the state vector evolves over time. Existing methods for state estimation cannot account for model-structure uncertainty. Worse still, not accounting for such uncertainty will introduce bias in estimates for the state. Although there has been some work in model-free state estimation to address such realities^4,23,24^, these approaches lose the inherent interpretability of handcrafted models.

We introduce a method for handling model-structure uncertainty in a manner that recovers the interpretability of handcrafted models. We do so by learning the motion model in the form of a set of symbolic differential equations simultaneously to a state estimate. This modelling choice enables state estimation in situations in which there are substantial modelling errors or the underlying dynamics are partially or completely unknown. This advancement is made possible by a new reparametrization trick for Markov Gaussian processes that we introduce in this work.

Numerical studies are presented for a range of test cases to illustrate the performance of the proposed approach. In practical situations in which modelling errors are present (even small errors), our method outperforms standard state-estimation techniques. Furthermore, our approach for state estimation with unknown governing equations allows analysts to discover missing terms in (or the entirety of) the governing equations using indirect observations. In Methods, we show that our approach outperforms state-of-the-art algorithms for governing-equation discovery, particularly in the low-data and high-noise regimes, and can be used in situations in which the entire state is not measurable. Finally, in cases in which interpretable forward models are not required, we show that our approach can be used to infer neural stochastic differential equations (SDEs) without relying on a forward solver in training.

## Results

### Problem statement

To mathematically define our problem statement, consider a noisy observation process of the form

1 $${y}_{t}=g({x}_{t},t)+\eta ,$$

in which ${y}_{t}\in {{\mathbb{R}}}^{d}$ is the observation vector at time instant *t*, ${x}_{t}\in {{\mathbb{R}}}^{D}$ is the latent state vector, $g:{{\mathbb{R}}}^{D}\times {\mathbb{R}}\to {{\mathbb{R}}}^{d}$ is the observation function and $\eta \in {{\mathbb{R}}}^{d}$ denotes observation noise.

Given the time-series dataset, ${\mathcal{D}}={\{({t}_{i},{y}_{{t}_{i}})\}}_{i=1}^{N}$, in which *t*_*i*_ ∈ [0, *T*] is the timestamp associated with the observation ${y}_{{t}_{i}}\in {{\mathbb{R}}}^{d}$, our goal is to infer

1. A well-calibrated estimate of the state vector, *x*_*t*_, over the time window [0, *T*], and
2. The equations governing the temporal evolution of the state.

### Related work

This work lies at the intersection between three areas of science: (1) state estimation; (2) discovery of governing equations from measurements; and (3) Bayesian inference over functions. We summarize some relevant work in these areas below.

The classical state-estimation problem in which the governing equations are known has been widely studied in the literature; see, for example, the texts by Barfoot^1^ and Särkkä and Svensson^2^. There is also a wide body of literature focusing on simultaneous state and parameter estimation; see, for example, refs. ^3,17–20^. These approaches are useful for problems in which the structure of the governing equations is known and the uncertainties are strictly parametric. Although model-free state-estimation techniques can be used in situations in which dynamics are unknown^4,23–25^, these techniques lose the interpretability of handcrafted models. A comparison of the assumptions made here to those made by existing methods is provided in Extended Data Table 1.

In terms of inferring interpretable governing equations, the special case in which all the states are measurable (that is, the observation function in equation (1) is the identity) has been extensively studied; see, for example, refs. ^26–28^. In 2011, Wang et al.^26^ showed how governing equations could be identified from full state measurements with a compressive-sensing approach using a dictionary of polynomials. Brunton et al.^27^ studied the problem of discovering governing equations from full state measurements using a compressive-sensing approach and demonstrated that this is a very powerful tool for solving a wide class of problems in computational science and engineering. In principle, this approach can be used with incomplete measurements using the notion of time-delay embedding^26^. However, it is challenging to get this approach to work well in practice owing to numerical issues; see appendix 4.5 of ref. ^27^.

The focus of this work is on the practical problem of estimating the state and the governing equations in which only indirect measurements are available.

### Stochastic variational inference

Here we tackle the problem of state estimation with unknown governing equations using the machinery of stochastic variational inference (SVI)^29^. SVI is a powerful approach for approximate Bayesian inference that converts the problem of generating samples from an unnormalized density function into an optimization problem involving maximization of a stochastic approximation of the evidence lower bound (ELBO). Variational inference has been applied to solve a wide variety of problems in machine learning^30–32^.

SVI typically requires four ingredients: (1) priors over variables of interest; (2) a parametrized approximate posterior; (3) a tractable expression for the ELBO that admits unbiased stochastic approximations; and (4) a method for approximating gradients of expectations in the ELBO.

**Fig. 1:**
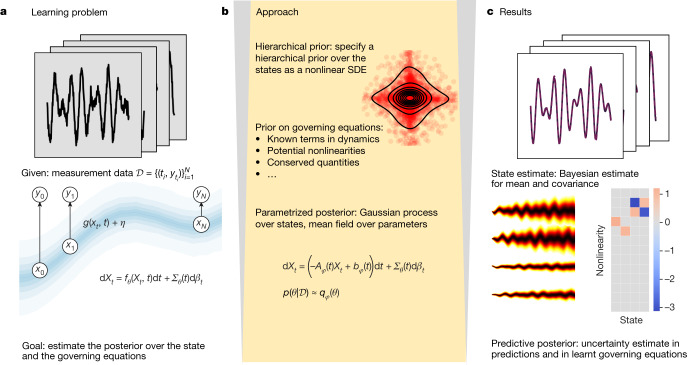
Main ideas. **a**, Given noisy time-series data and an observation model, our goal is to estimate the underlying state and any missing terms in the forward model. **b**, We specify a hierarchical prior over the state and the governing equations and then infer an approximate posterior over the state and forward model using SVI. **c**, We arrive with a state estimate, a method for generating forecasts with uncertainty estimates and a Bayesian estimate for missing terms in the governing equations.

In the context of the problem considered here, SVI cannot be applied in a straightforward manner because we seek to estimate a function, *x*_*t*_, given measurements of *y*_*t*_, rather than a finite-dimensional vector. Here we propose a reparametrization trick for approximating expectations under Markov Gaussian processes that enables us to tackle this challenge. A high-level summary of our approach is provided in Fig. 1 and we introduce the core ingredients of our methodology in the sections that follow.

### Priors

We place a hierarchical prior over the state in the form of an Itô SDE with an uncertain drift and diffusion function

2 $${\rm{d}}{X}_{t}={f}_{\theta }({X}_{t},t){\rm{d}}t+{\Sigma }_{\theta }(t){\rm{d}}{\beta }_{t},$$

in which ${f}_{\theta }:{{\mathbb{R}}}^{D}\times {\mathbb{R}}\to {{\mathbb{R}}}^{D}$ is the drift function, ${\Sigma }_{\theta }(t):{\mathbb{R}}\to {{\mathbb{R}}}^{D\times D}$ is the dispersion matrix, ${\beta }_{t}\in {{\mathbb{R}}}^{D}$ indicates Brownian motion with diffusion matrix *Q* and $\theta \in {{\mathbb{R}}}^{M+D}$ is a vector of parameters. The prior for the state captures our assumption that the structural form of the governing equations is partially or completely unknown.

To learn interpretable dynamics, it is often convenient to make the assumption that the drift function can be approximated by a sparse, linear combination of known basis functions

3 $${f}_{\theta }({x}_{t},t)={f}_{\varnothing }({x}_{t},t)+\mathop{\sum }\limits_{i=1}^{M}{\psi }_{i}({x}_{t},t){\theta }_{i},$$

in which ${f}_{\varnothing }:{{\mathbb{R}}}^{D}\times {\mathbb{R}}\to {{\mathbb{R}}}^{D}$ indicates the drift function of the known dynamics and ${\psi }_{i}:{{\mathbb{R}}}^{D}\times {\mathbb{R}}\to {{\mathbb{R}}}^{D}$ is the *i*th function from a predefined dictionary of basis functions. Often, this dictionary may include polynomials, trigonometric functions etc. We parametrize the dispersion matrix as ${\Sigma }_{\theta }={\Sigma }_{\varnothing }+{\rm{diag}}\left({\theta }_{M+1}^{2},{\theta }_{M+2}^{2},\ldots ,{\theta }_{M+D}^{2}\right)$, in which ${\Sigma }_{\varnothing }\in {{\mathbb{R}}}^{D\times D}$ refers to the known terms in the dispersion matrix.

As an aside, it is worth noting that it is straightforward to use more general parametrizations of the drift function, *f*_*θ*_. For instance, if we are interested in inferring Hamiltonians or more general energy cycles, we could parametrize *f*_*θ*_ by a Hamiltonian^33^ or generalized Hamiltonian^34^ neural network, respectively. Later, we provide examples to demonstrate the case when *f*_*θ*_ is parametrized by a fully connected neural network.

In alignment with the assumption of a sparse linear combination of basis functions and to indicate our preference that perturbations to the dispersion matrix be small or sparse if possible, we will make use of a sparsity-inducing horseshoe prior over the parameters, *θ* ∼ *p*(*θ*) (ref. ^35^).

We have now specified a hierarchical prior over the latent process we wish to infer, *X*|*θ* and *p*(*θ*). Here *X*|*θ* indicates the process defined by the SDE in equation (2) over sample paths in the interval [0, *T*] conditioned on a particular setting of the parameters. State estimation amounts to inferring the posterior process over *X* and inferring missing terms in the governing equations amounts to inferring the posterior over *θ*. For the special case in which the governing equations are known, the assumptions made in this work coincide with those made in continuous time-state estimation^1,2^.

### Variational distributions

To carry out SVI, we need to construct parametrized variational distributions for the state and the parameters in equations (2) and (3). We approximate the posterior process over the state as the solution to a linear SDE of the form

4 $${\rm{d}}{X}_{t}=(-{A}_{\varphi }(t){X}_{t}+{b}_{\varphi }(t)){\rm{d}}t+{\Sigma }_{\theta }{\rm{d}}{\beta }_{t},$$

in which ${A}_{\varphi }:{\mathbb{R}}\to {{\mathbb{R}}}^{D\times D}$ and ${b}_{\varphi }:{\mathbb{R}}\to {{\mathbb{R}}}^{D}$ are symmetric-matrix and vector-valued functions of time, respectively, *φ* is a vector of variational parameters, *Σ*_*θ*_d*β*_*t*_ is the same diffusion process defined in the prior and the initial condition is assumed to be Gaussian, ${X}_{0} \sim {\mathcal{N}}({m}_{0},{S}_{0})$.

Because the solution of a linear SDE is a Markov Gaussian process, we can parametrize the marginal statistics of the solution of equation (4) as ${q}_{\varphi }({x}_{t})={\mathcal{N}}\left({m}_{\varphi }(t),{S}_{\varphi }(t)\right)$, in which ${m}_{\varphi }:{\mathbb{R}}\to {{\mathbb{R}}}^{D}$ is the mean and ${S}_{\varphi }:{\mathbb{R}}\to {{\mathbb{R}}}^{D\times D}$ is the (marginal) covariance^36^. The marginal statistics satisfy the following system of ordinary differential equations (ODEs):

5 $${\dot{m}}_{\varphi }(t)=-\,{A}_{\varphi }(t){m}_{\varphi }(t)+{b}_{\varphi }(t)$$

6 $${\dot{S}}_{\varphi }(t)=-\,{A}_{\varphi }(t){S}_{\varphi }(t)-{S}_{\varphi }(t){A}_{\varphi }{(t)}^{T}+{\Sigma }_{\theta }Q{\Sigma }_{\theta }^{T}$$

in which *m*_*φ*_(0) = *m*_0_ and *S*_*φ*_(0) = *S*_0_.

To proceed further, we also need to define an approximate posterior for the parameters, *θ*, defined in equation (3). We used the log-normal parametrization from ref. ^37^, which we compactly denote by *q*_*φ*_(*θ*), in which again *φ* denotes the vector of variational parameters; see Methods for more details.

### ELBO

In SVI, the variational parameters are estimated by maximizing the ELBO, which is equivalent to minimizing the Kullback–Leibler (KL) divergence between the approximate posterior and the true, intractable posterior^29^. Here we propose a new reparametrization strategy that enables the variational parameters to be efficiently estimated without using a forward solver.

Letting ${{\mathbb{E}}}_{\widetilde{P}| \theta }\left[\cdot \right]$ indicate expectations under the prior SDE in equation (2), we can derive the following ELBO^38–40^

7 $$\log p({\mathcal{D}})=\log {{\mathbb{E}}}_{p(\theta )}\,\left[\,{{\mathbb{E}}}_{\widetilde{P}| \theta }\,\left[\,\mathop{\prod }\limits_{i=1}^{N}p({y}_{{t}_{i}}| {x}_{{t}_{i}})\right]\right]$$

8 $$\begin{array}{l}\,\ge \mathop{\sum }\limits_{i=1}^{N}{{\mathbb{E}}}_{{q}_{\varphi }({x}_{{t}_{i}})}[\log p({y}_{{t}_{i}}| {x}_{{t}_{i}})]\\ \,-\frac{1}{2}{\int }_{0}^{T}{{\mathbb{E}}}_{{q}_{\varphi }({x}_{t}){q}_{\varphi }(\theta )}\,[\,\parallel r({x}_{t},t,\theta ,\varphi ){\parallel }_{{\Sigma }_{\theta }Q{\Sigma }_{\theta }^{T}}^{2}]{\rm{d}}t\\ \,-\,{D}_{{\rm{KL}}}({q}_{\varphi }(\theta )\parallel p(\theta ))={\rm{ELBO}}(\varphi ),\end{array}$$

in which *r*(*x*_*t*_, *θ*, *φ*) = −*A*_*φ*_(*t*)*x*_*t*_ + *b*_*φ*_(*t*) − *f*_*θ*_(*x*_*t*_, *t*) is the drift residual, $| | v| {| }_{{\Sigma }_{\theta }Q{\Sigma }_{\theta }^{T}}^{2}={v}^{T}{\left({\Sigma }_{\theta }Q{\Sigma }_{\theta }^{T}\right)}^{-1}v$ and *D*_KL_(*q*_*φ*_(*θ*)||*p*(*θ*)) indicates the KL divergence between *q*_*φ*_(*θ*) and *p*(*θ*).

Maximizing equation (8) with respect to the parameters of the variational distributions, *φ*, would provide an approximate state estimate, *q*_*φ*_(*x*_*t*_), and an estimate for the posterior distribution over the parameters, *q*_*φ*_(*θ*). Unfortunately, maximizing this ELBO is computationally challenging because the first two terms depend on expectations with respect to *q*_*φ*_(*x*_*t*_), the current state estimate. Archambeau et al.^38,39^ explored maximizing equation (8) subject to the differential equality constraints in equations (5) and (6) in the context of dynamic data assimilation. This approach requires solving 2(*D* + *D*^2^) ODEs at each optimization iteration. Solving ODEs as part of an optimization procedure is computationally challenging because: (1) gradient-based updates to the variational parameters can cause the ODEs to become extremely stiff mid-optimization, causing the computational cost of an ODE solve to explode, and (2) ODE solvers are inherently iterative sequential methods, making them poorly suited to modern parallel-computing hardware.

Here we introduce a reparametrization for expectations in equation (8) with respect to *q*_*φ*_(*x*_*t*_) that replaces the need for an ODE forward solver with a stochastic approximation that can be evaluated in an embarrassingly parallel fashion. This eliminates the practical challenges associated with maximizing equation (8). Our main theoretical result takes the form

9 $$\begin{array}{l}{\rm{ELBO}}(\varphi )\,=\,\mathop{\sum }\limits_{i=1}^{N}{{\mathbb{E}}}_{{\mathcal{N}}({m}_{\varphi }({t}_{i}),{S}_{\varphi }({t}_{i}))}[\log p({y}_{{t}_{i}}| {x}_{{t}_{i}})]\\ \,\,\,-\,\frac{1}{2}{\int }_{0}^{T}{{\mathbb{E}}}_{{\mathcal{N}}({m}_{\varphi }(t),{S}_{\varphi }(t)){q}_{\varphi }(\theta )}\,[\,\parallel r({x}_{t},t,\theta ,\varphi ){\parallel }_{{\Sigma }_{\theta }Q{\Sigma }_{\theta }^{T}}^{2}]{\rm{d}}t\\ \,\,\,-\,{D}_{{\rm{KL}}}({q}_{\varphi }(\theta )\parallel p(\theta )),\end{array}$$

in which

10 $$\begin{array}{l}r({x}_{t},t,\theta ,\varphi )\,=\,{{\rm{vec}}}^{-1}({({S}_{\varphi }(t)\oplus {S}_{\varphi }(t))}^{-1}{\rm{vec}}({\Sigma }_{\theta }Q{\Sigma }_{\theta }^{T}-{\dot{S}}_{\varphi }(t)))\\ \,\,\,({m}_{\varphi }(t)-{x}_{t})+{\dot{m}}_{\varphi }(t)-{f}_{\theta }({x}_{t},t),\end{array}$$

denotes the drift residual reparametrized in terms of *m*_*φ*_ and *S*_*φ*_ (for a derivation, see Methods), ⊕ indicates the Kronecker sum, ${\rm{vec}}:{{\mathbb{R}}}^{D\times D}\to {{\mathbb{R}}}^{{D}^{2}}$ maps a matrix into a vector by stacking columns and ${{\rm{vec}}}^{-1}:{{\mathbb{R}}}^{{D}^{2}}\to {{\mathbb{R}}}^{D\times D}$ unstacks a vector such that vec^−1^(vec(*C*)) = $C\,{\rm{\forall }}\,C\in {{\mathbb{R}}}^{D\times D}$.

Recall that, in the original ELBO (equation (8)), the first two terms contained expectations with respect to the marginal statistics of the solution of the SDE (equation (4)), *q*_*φ*_(*x*_*t*_), and approximating these expectations required the use of a forward solver. In this reparametrized ELBO (equation (9)), expectations are taken with respect to the Gaussian, ${\mathcal{N}}\left({m}_{\varphi }(t),{S}_{\varphi }(t)\right)$. It is worth noting that, as we directly parametrize *m*_*φ*_ and *S*_*φ*_ instead of *A*_*φ*_ and *b*_*φ*_, it is feasible to estimate all expectations without running a forward ODE solver. Notably, we no longer need to solve any ODEs; we only need to construct stochastic approximations of the terms in the ELBO. Such stochastic approximations are easy to compute using modern parallel-computing hardware. It is this new development that enables us to perform approximate Bayesian inference over both the state and the unknown governing equations.

In Methods, we describe how to parametrize *m*_*φ*_ and *S*_*φ*_ and how to optimize a stochastic approximation of the ELBO (equation (9)). Having maximized the ELBO, we are left with an approximation to the posterior over the state, ${q}_{\varphi }({x}_{t})={\mathcal{N}}\left({m}_{\varphi }(t),{S}_{\varphi }(t)\right)$, and an approximate posterior over the parameters defining the governing equations, *q*_*φ*_(*θ*), that can be used to make probabilistic forecasts and perform further analysis. We henceforth refer to our method as SVI for state estimation (SVISE). Our method was implemented in PyTorch^41^. In the following sections, we provide some examples of SVISE applied to problems in state estimation and governing-equation discovery.

### Example 1: state estimation with known motion model

In this section, we compare our method for state estimation with unknown governing equations to the particle filter (PF)^42,43^ when the form of the underlying motion model is known exactly. The PF was chosen as the baseline for comparison as it is a fully Bayesian method capable of handling highly nonlinear systems. Both methods were provided with complete knowledge of the underlying dynamics and observation model. We performed this comparison on six benchmark problems and a detailed description of the experiment design is provided in Methods.

**Fig. 2:**
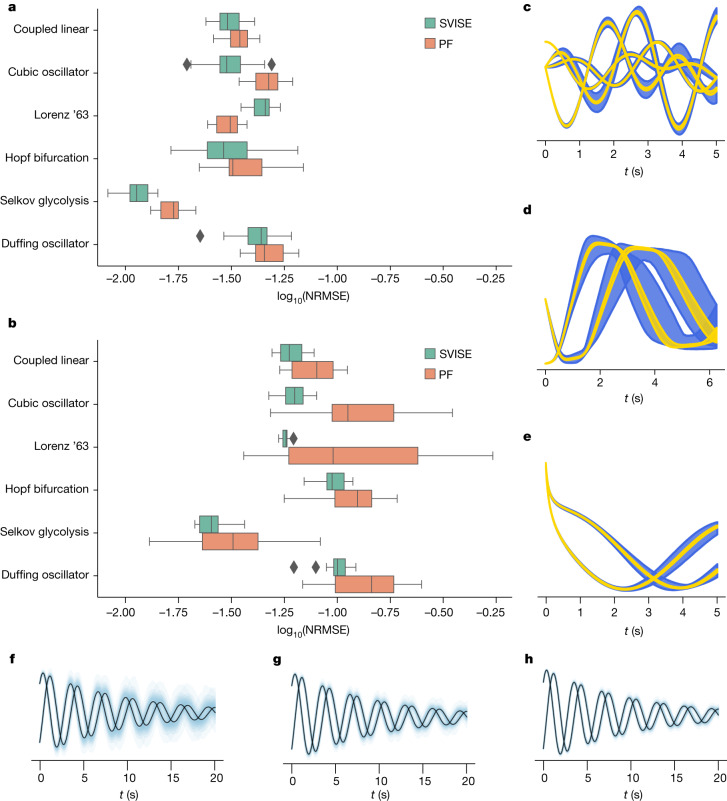
Benchmarking summary with 20 independent trials per system. **a**, State-estimation results with perfect knowledge of the dynamics. **b**, State-estimation results with minor corruption of the dynamics. **c**–**e**, Before and after adding a small probabilistic corruption for the coupled linear oscillator (**c**), the cubic oscillator (**d**) and the Hopf bifurcation (**e**) (10–90th quantiles shown). **f**–**h**, Probabilistic forecast with 64 (**f**), 512 (**g**) and 1,024 (**h**) data points. We see that our method (SVISE) performs similarly to the PF when the governing equations are known exactly. In the presence of only mild modelling error, our method outperforms the PF on average. Moreover, after training, our method enables us to make probabilistic forecasts. Expectedly, the predictive posterior shrinks as the amount of data is increased.

The results are summarized in Fig. 2a. We found that our method provided results comparable with the PF in terms of providing an estimate for the mean of the state. We believe that minor differences in performance are because of our choice of basis functions. Clearly there is an opportunity to perform model selection in terms of the basis-function design if desired.

### Example 2: state estimation with modelling errors

In this section, we examine the performance of our method on the same suite of benchmarks when a small modelling error is introduced. We introduce a small state-dependent corruption to the governing equations and compare the performance of SVISE with the PF. Unlike the previous set of examples, we do not provide our method with access to knowledge of the dynamics (that is, we set ${f}_{\varnothing }=0$ and ${\Sigma }_{\varnothing }=0$), so that we are required to learn the dynamics as well as a state estimate. The PF is given access to the uncorrupted dynamics, along with the exact form of the diffusion process.

The specific form of the corruption is chosen so that the form of the governing equations are, on average, the same as in the previous section and so that any particular realization of a corruption could plausibly be mistaken for further process noise rather than model misspecification; see Fig. 2c–e. Further details can be found in Methods.

Because the PF is given the exact form of the diffusion process, we believe that upcoming results represent an overestimate for the PF performance. Despite this advantage, we find that our method now, on average, outperforms the PF in the presence of this mild corruption to the dynamics; see Fig. 2b. As discussed previously, there are many real-world systems for which modelling errors are an unavoidable reality. This work can be used in such circumstances to potentially improve on state-estimation performance.

As was previously discussed, a key differentiating feature of our method compared with model-free state-estimation techniques is that we are learning symbolic differential equations for the motion model. Looking to Fig. 2f,g,h, we see that our approach can be used to make probabilistic forecasts after state estimation has concluded.

### Example 3: governing-equation discovery with neural SDEs

When the state is low dimensional, or the state is spatially extended, it is possible to make use of dictionaries of polynomial basis functions to infer symbolic SDEs simultaneously to a state estimate. In Methods, we provide three numerical studies demonstrating the application of our approach to symbolic-governing-equation discovery. We show that our approach offers superior performance to state-of-the-art algorithms for the sparse identification of nonlinear dynamics in the presence of substantial noise or a lack of data. Also, we demonstrate scalability by showing that our approach can be used to infer symbolic governing equations for a spatially extended system with 1,024 states. Finally, we show that our approach can be used to infer symbolic governing equations for second-order systems using only position measurements.

Unfortunately, dictionaries of polynomial basis functions become prohibitively large even for moderately high-dimensional systems. In such situations, a reasonable alternative to inferring symbolic differential equations is to parametrize the drift function by a neural network. In Methods, we present numerical studies on a binary-black-hole problem that involves inferring a neural SDE using a nonlinear observation function. In the example that follows, we consider a fluid-dynamics problem with a high-dimensional state space to illustrate how our approach can be combined with off-the-shelf dimensionality-reduction algorithms to infer probabilistic reduced-order models (ROMs) in the format of latent neural SDEs. The main idea of ROMs is to infer a mapping that enables the original high-dimensional system to be transformed into a low-dimensional system that is computationally cheaper to solve^44^. The approach we take is aligned with previous works in the ROMs literature that infer lower-dimensional differential equations on a predefined manifold^45,46^.

**Fig. 3:**
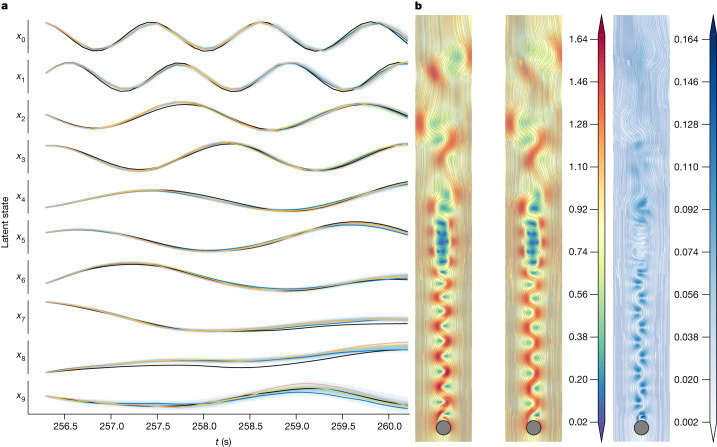
Flow behind a cylinder ROM problem. **a**, Predictions for the first ten latent states over the testing time interval *t* = [256.2, 260.2]. The black lines show the test states and the coloured lines indicate samples from the predictive posterior. **b**, Velocity magnitude and flow lines at *t* = 260.2. Test (left), mean prediction (centre) and standard deviation (right). In this example, we train a neural SDE on trajectories projected onto the POD basis to construct a ROM. Although a neural SDE is less interpretable than a symbolic model, it is useful in cases in which the state is either (1) high dimensional or (2) it is not clear which basis functions might be appropriate for the problem at hand. We see that the error bars are higher in regions in which the mean prediction seems to differ from the test velocity.

As a case study, we considered the challenging problem of inferring latent differential equations for fluid flow past a circular cylinder with a Reynolds number of 2,000. We model this problem using a spatial discretization of the two-dimensional incompressible Navier–Stokes equation with 596,602 states. We train a neural SDE on 38 latent states. These latent states were constructed by projecting the data onto the top 38 proper orthogonal decomposition (POD) modes to capture 90% of the total variance. More details on the numerical study design are provided in Methods. It can be seen from Fig. 3 that our approach provides accurate probabilistic predictions of the flow field.

## Discussion

We have presented a method for state estimation that enables the treatment of real-world problems in which there are notable modelling errors or the underlying governing equations are completely or partially unknown. This is an important class of problems that has thus far defied a computationally tractable, statistically rigorous solution. We have also provided results for governing-equation discovery for a variety of challenging problems. The results indicate that our approach can outperform state-of-the-art algorithms for governing-equation discovery often by more than an order of magnitude; see Methods for details. It is worth reiterating that many governing-equation learning algorithms only provide point parameter estimates^27^. As demonstrated in Fig. 2f–h, our method enables Bayesian governing-equation identification, thereby allowing for probabilistic forecasts to be made.

Moreover, in cases in which specifying a dictionary of basis functions is not feasible (such as when the state space is high dimensional or it is not clear what basis functions are appropriate), we have shown that it is possible to infer the missing dynamics in the form of a neural network. It is worth noting here that our approach enables neural SDEs to be trained without using a computationally expensive forward solver and adjoint sensitivity calculations.

These results are made possible by a reparametrization trick for Markov Gaussian processes that we introduce here. We believe that there is substantial room for future work in applying this technique for performing variational inference over stochastic processes outside the state-estimation domain. Potential application areas include multiphysics ROM problems, state estimation with unknown observation models, stochastic closure modelling^47^ and inferring latent forcing models^48^. Also, there is room for using more expressive approximations to the posterior over the parameters to better approximate uncertainty. Finally, there has been a recent resurgence of interest in using SDEs for building complex generative models^40^ and we expect that our approach could be used to gain deeper insights into the statistical underpinnings of such models.

## Methods

### Reparametrized drift residual

Recall that, given a linear SDE, d*X*_*t*_ = (−*A*_*φ*_(*t*)*X*_*t*_ + *b*_*φ*_(*t*))d*t* + *Σ*_*θ*_d*β*_*t*_, with initial condition ${X}_{0} \sim {\mathcal{N}}({m}_{0},{S}_{0})$, we can solve for the marginal statistics of this SDE, ${q}_{\varphi }({x}_{t})={\mathcal{N}}\left({m}_{\varphi }(t),{S}_{\varphi }(t)\right)$, by solving the ODEs in equations (5) and (6). Given *S*_*φ*_ and ${\dot{S}}_{\varphi }$, we notice that equation (6) is a set of Lyapunov equations in terms of the symmetric matrix *A*_*φ*_. We can rearrange this set of equations to express *A*_*φ*_ and *b*_*φ*_ explicitly in terms of *m*_*φ*_ and *S*_*φ*_

11 $${\rm{vec}}({A}_{\varphi }(t))={({S}_{\varphi }(t)\oplus {S}_{\varphi }(t))}^{-1}{\rm{vec}}\left({\Sigma }_{\theta }Q{\Sigma }_{\theta }^{T}-{\dot{S}}_{\varphi }(t)\right),$$

12 $${b}_{\varphi }(t)=\dot{m}(t)+{A}_{\varphi }(t){m}_{\varphi }(t).$$

*S*_*φ*_(*t*) ⊕ *S*_*φ*_(*t*)(*S*_*φ*_(*t*) ⊕ *S*_*φ*_(*t*) = *S*_*φ*_(*t*) ⊗ *I* + *I* ⊗ *S*_*φ*_(*t*) is the Kronecker sum, in which ⊗ indicates the standard Kronecker product.) is guaranteed to be invertible because *S*_*φ*_ is a valid covariance matrix. Using equations (11) and (12), the drift residual can be rewritten as follows:

13 $$r({x}_{t},t,\theta ,\varphi )=-\,{A}_{\varphi }(t){x}_{t}+{b}_{\varphi }(t)-{f}_{\theta }({x}_{t},t),$$

14 $$=\,{A}_{\varphi }(t)({m}_{\varphi }(t)-{x}_{t})+\dot{m}(t)-{f}_{\theta }({x}_{t},t),$$

15 $$\begin{array}{l}=\,{{\rm{vec}}}^{-1}\left({\left({S}_{\varphi }(t)\oplus {S}_{\varphi }(t)\right)}^{-1}\,{\rm{vec}}\left({\Sigma }_{\theta }Q{\Sigma }_{\theta }^{T}-{\dot{S}}_{\varphi }(t)\right)\right)\left({m}_{\varphi }(t)-{x}_{t}\right)\\ \,\,+\,{\dot{m}}_{\varphi }(t)-{f}_{\theta }({x}_{t},t),\end{array}$$

Substituting this into the expression for the ELBO (equation (8)) yields the final form of the reparametrized ELBO (equation (9)).

### Parametrization of *m*_*φ*_ and *S*_*φ*_

We parametrize *m*_*φ*_ using radial-basis-function models of the form *m*_*φ*_(*t*) = (*k*^(1)^(*t*, *τ*) ⊗ *I*)*φ*^(1)^, in which $${\varphi }^{(1)}\in {{\mathbb{R}}}^{pD}$$ is the vector of weights associated with the approximation for the mean, $${k}^{(1)}(t,\tau )\,=$$$$[1\,{k}^{(1)}(t,{t}_{1})\,\ldots \,{k}^{(1)}(t,{t}_{p-1})]\in {{\mathbb{R}}}^{1\times p}$$ is the vector of basis functions evaluated at *t* and *τ* = {*t*_1_,…, *t*_*p*−1_} is the set of basis function centres. We chose each *k*^(*j*)^ to be the Matérn 5/2 kernel^49^ centred at *t*_*i*_ ∈ [0, *T*],

$${k}^{(j)}(t,{t}_{i})={\sigma }_{j}^{2}\left(1+\sqrt{5}r(t,{t}_{i})/{{\ell }}_{j}+5r{(t,{t}_{i})}^{2}/(3{{\ell }}_{j}^{2})\right)\exp \left(-\sqrt{5}r(t,{t}_{i})/{{\ell }}_{j}\right),$$

in which *σ*_*j*_ > 0 and *ℓ*_*j*_ > 0 are the tunable scale and length scales associated with the basis functions, respectively, and *r*(*t*, *t*_*i*_) = |*κ*(*t*) − *κ*(*t*_*i*_)| with $\kappa (t)=T-T{\left(1-{(t/T)}^{\alpha }\right)}^{\beta }$ denoting the Kumaraswamy warping function with two positive tunable parameters *α* and *β* (ref. ^50^). In all experiments, unless otherwise noted, we used 200 evenly spaced basis functions within the time interval [0, *T*].

When dealing with systems whose dynamics can be characterized in a low-dimensional state space, we use a full-rank, dense, spectral parametrization of the state covariance matrix *S*_*φ*_(*t*) = *R*_*φ*_(*t*)*Λ*_*φ*_(*t*)*R*_*φ*_(*t*)^*T*^, in which *R*_*φ*_(*t*) is an orthonormal matrix and *Λ*_*φ*_(*t*) is a diagonal matrix with all positive elements that we parametrize as $${R}_{\varphi }(t)\,=$$$$\exp ({(({k}^{(2)}(t,\tau )\otimes I){\varphi }^{(2)})}^{\wedge })$$ and $${\varLambda }_{\varphi }(t)={\rm{s}}{\rm{o}}{\rm{f}}{\rm{t}}{\rm{p}}{\rm{l}}{\rm{u}}{\rm{s}}\,({\rm{d}}{\rm{i}}{\rm{a}}{\rm{g}}(({k}^{(3)}(t,\tau )\otimes I){\varphi }^{(3)}))$$, in which $${\varphi }^{(2)}\in {{\mathbb{R}}}^{pD(D-1)/2}$$ is the vector of weights associated with *R*_*φ*_, $${\varphi }^{(3)}\in {{\mathbb{R}}}^{pD}$$ is the vector of weights associated with *Λ*_*φ*_, $${k}^{(j)}(t,\tau )\,=$$$$[1\,{k}^{(j)}(t,{t}_{1})\,\ldots \,{k}^{(j)}(t,{t}_{p-1})]\in {{\mathbb{R}}}^{1\times p}$$ is the vector of basis functions evaluated at *t*, $${(\cdot )}^{\wedge }:{{\mathbb{R}}}^{D(D-1)/2}\to {{\mathbb{R}}}^{D\times D}$$ is an operator that turns a vector of length *D*(*D* − 1)/2 into a skew-symmetric matrix (notation from ref. ^1^), $$\exp :{{\mathbb{R}}}^{D\times D}\to {{\mathbb{R}}}^{D\times D}$$ indicates the matrix exponential, $${\rm{diag}}:{{\mathbb{R}}}^{D}\to {{\mathbb{R}}}^{D\times D}$$ turns a vector of length *D* into a diagonal matrix and $${\rm{softplus}}:{\mathbb{R}}\to {\mathbb{R}}$$ is the Softplus function that acts element wise. This parametrization requires $${\mathcal{O}}\left({D}^{3}\right)$$ time to compute.

Under this parametrization, we can solve equation (6) for *A*_*φ*_ by first solving the system of linear equations

16 $$({\varLambda }_{\varphi }(t)\oplus {\varLambda }_{\varphi }(t)){\rm{v}}{\rm{e}}{\rm{c}}({B}_{\varphi }(t))={\rm{v}}{\rm{e}}{\rm{c}}({R}_{\varphi }{(t)}^{T}({\Sigma }_{\theta }Q{\Sigma }_{\theta }^{T}-{\dot{S}}_{\varphi }(t)){R}_{\varphi }(t))$$

and then computing *A*_*φ*_(*t*) = *R*_*φ*_(*t*)*B*_*φ*_(*t*)*R*_*φ*_(*t*)^*T*^. This result follows from the application of the Bartels–Stewart algorithm^51^.

To summarize, this parametrization allows for complete flexibility in terms of its ability to approximate symmetric positive-definite matrices; however, this flexibility comes at the cost of scaling as ${\mathcal{O}}\left({D}^{3}\right)$ owing to the matrix–matrix products. This computational cost makes it only applicable for use with relatively low-dimensional systems.

When dealing with high-dimensional dynamical systems, as is often the case in the geosciences for example, we propose parametrizing *S*_*φ*_ by a purely diagonal covariance matrix, that is, *S*_*φ*_(*t*) = *Λ*_*φ*_(*t*), in which *Λ*_*φ*_(*t*) is parametrized as described previously for the spectral parametrization. If we also restrict ${\Sigma }_{\theta }Q{\Sigma }_{\theta }^{T}$ to be diagonal, we have ${A}_{\varphi }(t)=\frac{1}{2}{S}_{\varphi }{(t)}^{-1}\left({\Sigma }_{\theta }Q{\Sigma }_{\theta }^{T}-{\dot{S}}_{\varphi }(t)\right)$. The dimensionality of this parametrization scales linearly in the dimension of the state, ${\mathcal{O}}(D)$, making state estimation with unknown governing equations possible in extremely high dimensions.

We use the diagonal parametrization for the reduced-order modelling problem in the main text as well as the upcoming studies of our approach applied to symbolic-governing-equation discovery for high-dimensional spatially extended systems, as well as the example of our approach applied to the binary black hole. For all other numerical studies, we use the full spectral parametrization.

### Maximization of the ELBO

We approximate all gradients of expectations using the standard reparametrization trick^30^. If the observation function is linear, we can exactly compute the expected log-likelihood, as is described in an upcoming section. We used the so-called hybrid simulator based on Gaussian quadrature^52^ to estimate the integral over time with respect to the drift residual. This integration scheme ensures that approximations to the ELBO gradient remain unbiased while providing lower variance than standard Monte Carlo. In the case that the number of data points is large, stochastic gradient ascent can be used to maximize the objective. We used Adam^53^ to optimize the ELBO with respect to the variational parameters.

Putting these computational ingredients together, and letting each ${x}_{t}^{(\,j)},{\theta }^{(j)} \sim {\mathcal{N}}({m}_{\varphi }(t),{S}_{\varphi }(t)){q}_{\varphi }(\theta )$ be samples from the variational distributions drawn using the reparametrization trick, we can write stochastic approximations to the first term in the ELBO as

17 $$\mathop{\sum }\limits_{i=1}^{N}{{\mathbb{E}}}_{{\mathcal{N}}({m}_{\varphi }({t}_{i}),{S}_{\varphi }({t}_{i}))}\left[\log p({y}_{{t}_{i}}| {x}_{{t}_{i}})\right]\approx \frac{N}{BJ}\mathop{\sum }\limits_{i=1}^{B}\mathop{\sum }\limits_{j=1}^{J}\log p({y}_{{t}_{i}}| {x}_{{t}_{i}}^{(\,j)}),$$

in which each *i* has been uniformly sampled from the set {1, 2,…, *N*}. Following ref. ^52^ and letting $I(t)=-\,\frac{1}{2J}{\sum }_{j=1}^{J}| | r\left({x}_{t}^{(\,j)},t,{\theta }^{(j)},\varphi \right)| {| }_{{\Sigma }_{{\theta }^{(j)}}Q{\Sigma }_{{\theta }^{(j)}}^{T}}^{2}$, we can write a stochastic approximation to the second term in the ELBO as

18 $$\begin{array}{l}-\frac{1}{2}{\int }_{0}^{T}{{\mathbb{E}}}_{{\mathcal{N}}({m}_{\varphi }(t),{S}_{\varphi }(t)){q}_{\varphi }(\theta )}[| | r({x}_{t},t,\theta ,\varphi )| {| }_{{\Sigma }_{\theta }Q{\Sigma }_{\theta }^{T}}^{2}]{\rm{d}}t\\ \approx \,\frac{1}{K}\mathop{\sum }\limits_{k=1}^{K}\left(TI({t}_{k})-T{P}_{M-1}({t}_{k})+\mathop{\sum }\limits_{i=1}^{M}{\omega }_{i}I({t}_{i})\right)\end{array}$$

in which ${\omega }_{i}\in {\mathbb{R}}$ is the *i*th quadrature weight associated with the Gauss–Legendre quadrature rule^54^ and *P*_*M*−1_ is the *(M* − 1)-degree polynomial interpolant of *I*(*t*) constructed by matching the value of *I*(*t*) at the quadrature nodes. Recall that we have selected *q*_*φ*_(*θ*) and *p*(*θ*) such that we can write the KL divergence between the two distributions in closed form.

To demonstrate the robustness of our approach, we kept hyperparameters constant across all experiments unless otherwise specified. We chose *B* = min(128, number of data points), *J* = 32, *K* = 26 and *M* = 102. We chose a learning rate of 10^−3^ for all parameters related to *q*_*φ*_(*x*_*t*_) and 10^−2^ for all other parameters; for the Lorenz ’96 problem, we chose learning rates 10^−2^ and 10^−1^, respectively. We trained every model for 20,000 iterations; for the Lorenz ’96 problem, we trained for 5,000 iterations. We used 5,000 warm-up iterations (1,250 for the Lorenz ’96 problem) wherein the KL divergence term is scaled by a constant factor from 0 to 1, increasing every iteration at a linear rate^55^. We decayed the learning rate by multiplying the starting learning rate by 0.9 every 2,500 iterations (625 for the Lorenz ’96 problem). Although these were the hyperparameter settings we chose for all experiments here, we would like to emphasize that this is just one setting for the hyperparameters that worked well consistently. Careful tuning for individual experiments can probably improve performance and or decrease training time.

### Initialization

We found that good initialization of the radial-basis-function models used to parametrize *m*_*φ*_ and *S*_*φ*_ could greatly improve convergence. This is to be expected, given that we are trying to maximize a highly nonconvex objective. For the constant basis function, we initialized the weight to 0 in the mean-function and orthogonal-matrix parametrizations. For the eigenvalue parametrization (*Λ*_*φ*_), we initialized this weight to approximately −2.5. We also added 10^−6^ to the eigenvalue matrix to help ensure that the optimizer stayed away from extremely degenerate regions of the optimization space in the early stages. Initializing the eigenvalues to be small at the start of the optimization procedure helped to avoid convergence to a poor local minima wherein the model finds that the data were generated by a pure random-walk process. For the scale parameter, *σ*_*j*_, we always initialized to 1. For the mean function, we initialized the remaining weights in *φ*^(1)^ by minimizing the least-squares error with *ℓ*_2_ regularization on the training data. We weighted the *ℓ*_2_ regularization term by 10^−1^. We initialized the length scale with a grid search over the length scales from the set {10^−1^, 1/2, 1, 10} using fivefold cross-validation. For the orthogonal matrix, we initialized the weights to a small positive constant, 10^−6^. For the sparse approximation to the drift function, we initialized the weights by minimizing the *ℓ*_2_ regularized least-squares error between the derivatives from the initialized mean function and the drift-function model at the training timestamps. Again, we weighted the *ℓ*_2_ regularization term by 10^−1^.

### Normalization of drift-function features

When training sparse linear models, we found that normalizing the drift-function features dynamically in training could make convergence to a good minima more consistent. In this work, we assumed that we were only interested in time-independent features and that we were using the same features in each dimension. In this case, we can write, *f*_*θ*_(*x*_*t*_, *t*) = (*ψ*(*x*_*t*_) ⊗ *I*)*θ*, in which $\psi :{{\mathbb{R}}}^{D}\to {{\mathbb{R}}}^{1\times M}$ returns a row vector of features and $\theta \in {{\mathbb{R}}}^{DM}$ is a vector of parameters. Let *ψ*(*x*) be the basis functions evaluated at a batch of inputs. During training, each time we compute *ψ*(*x*), we normalize on the basis of the running variance, that is,

19 $$\psi (x)\leftarrow \frac{\psi (x)}{\sqrt{{\rm{Var}}[\psi (x)]+{\epsilon }}},$$

in which Var[*ψ*(*x*)] is the running variance of the output features and *ϵ* > 0 is a constant we choose to be 10^−5^. The running variance is updated according to the rule Var[*ψ*(*x*)]_new_ = (1 − *μ*) × Var[*ψ*(*x*)]_old_ + *μ* × Var[*ψ*(*x*)]_est_, in which Var[*ψ*(*x*)]_est_ is the estimated variance for the current batch of inputs and the momentum, *μ*, was set to 0.1. The constant basis function *ψ*(*x*) = 1 was not normalized. This is very similar to the batch-normalization^56^ implementation provided in PyTorch^41^. The running variance was initialized using the variance of the inputs at the data timestamps using the initialized mean approximation.

### KL divergence for the half-Cauchy prior

This section summarizes the parametrization defined by ref. ^37^ that is used to estimate the posterior over the parameters in equation (3) when performing symbolic-governing-equation discovery. This is a useful parametrization as it allows us to easily sample from the approximate posterior and express the KL divergence between the approximate posterior and the prior in closed form.

Recall that the prior for the parameters is written as *p*(*θ*), in which $\theta \in {{\mathbb{R}}}^{M+D}$, *M* is the number of basis functions in the dictionary and *D* is the dimension of the state. We express the full hierarchical prior as ${\theta }_{i}={\widetilde{\theta }}_{i}\sqrt{{s}_{a}{s}_{b}\,{\alpha }_{i}\,{\beta }_{i}}$, in which

20 $${\widetilde{\theta }}_{i} \sim {\mathcal{N}}(0,1),\,{s}_{a} \sim {\mathcal{G}}(0.5,{\tau }_{0}^{2}),\,{s}_{b} \sim {\mathcal{IG}}(0.5,1),$$

21 $${\alpha }_{i} \sim {\mathcal{G}}(0.5,1)\,{\rm{and}}\,{\beta }_{i} \sim {\mathcal{IG}}(0.5,1).$$

Here ${\mathcal{G}}$ and ${\mathcal{IG}}$ denote the Gamma and inverse Gamma distributions, respectively, and *τ*_0_ is a small positive constant chosen by the user, typically ${\mathcal{O}}(1{0}^{-5})-{\mathcal{O}}(1{0}^{-7})$. The product $z=\sqrt{{s}_{a}\,{s}_{b}}$ corresponds to a half-Cauchy distribution on *z*. The idea behind the prior is ‘global–local’ shrinkage. Here *s*_*a*_ and *s*_*b*_ are ‘global’ scales that encourage all parameters to be small and *α*_*i*_ and *β*_*i*_ are local scales that allow the corresponding parameter to remain unconstrained.

Having specified the prior, we must now specify an approximate posterior. Following ref. ^37^, we make use of a mean-field assumption, approximating the posterior over the shrinkage parameters using log-normal distributions. The approximate posterior over all parameters can be expressed as the product

22 $${q}_{\varphi }(\theta )={q}_{\varphi }({s}_{a},{s}_{b})\mathop{\prod }\limits_{i=1}^{M+D}{q}_{\varphi }({\alpha }_{i},{\beta }_{i}){q}_{\varphi }({\widetilde{\theta }}_{i}),$$

in which *φ* is the vector of variational parameters corresponding to the distributions

23 $${q}_{\varphi }({s}_{a},{s}_{b})={\mathcal{LN}}({s}_{a}| {\mu }_{{s}_{a}},{\sigma }_{{s}_{a}}^{2}){\mathcal{LN}}({s}_{b}| {\mu }_{{s}_{b}},{\sigma }_{{s}_{b}}^{2});$$

24 $${q}_{\varphi }({\alpha }_{i},{\beta }_{i})={\mathcal{LN}}({\alpha }_{i}| {\mu }_{{\alpha }_{i}},{\sigma }_{{\alpha }_{i}}^{2}){\mathcal{LN}}({\beta }_{i}| {\mu }_{{\beta }_{i}},{\sigma }_{{\beta }_{i}}^{2});$$

25 $${q}_{\varphi }({\widetilde{\theta }}_{i})={\mathcal{N}}({\widetilde{\theta }}_{i}| {\mu }_{{\widetilde{\theta }}_{i}},{\sigma }_{{\widetilde{\theta }}_{i}}^{2}).$$

Given this choice of prior and posterior, the KL divergence between the approximate posterior and the prior factorizes as follows:

26 $${D}_{{\rm{KL}}}({q}_{\varphi }(\theta )| | p(\theta ))={D}_{{\rm{KL}}}({q}_{\varphi }({s}_{b})| | p({s}_{b}))+{D}_{{\rm{KL}}}({q}_{\varphi }(\alpha )| | p(\alpha ))+$$

27 $${D}_{{\rm{KL}}}({q}_{\varphi }(\beta )| | p(\beta ))+{D}_{{\rm{KL}}}({q}_{\varphi }(\widetilde{\theta })| | p(\widetilde{\theta })).$$

We can write each term in the KL divergence between the approximate posterior and the prior as

28 $$\begin{array}{l}{D}_{{\rm{KL}}}({q}_{\varphi }({s}_{b})| | p({s}_{b}))=\exp \left(\frac{1}{2}{\sigma }_{{s}_{b}}^{2}-{\mu }_{{s}_{b}}\right)\\ \,\,\,\,-\,\frac{1}{2}\left(-{\mu }_{{s}_{b}}+2\,\log {\sigma }_{{s}_{a}}+1+\log \,2\right),\end{array}$$

29 $$\begin{array}{l}{D}_{{\rm{KL}}}({q}_{\varphi }(\alpha )| | p(\alpha ))=\mathop{\sum }\limits_{i=1}^{M+D}\left(\exp \left({\mu }_{{\alpha }_{i}}+\frac{1}{2}{\sigma }_{{\alpha }_{i}}^{2}\right)\right.\\ \,\,\,\,\left.-\,\frac{1}{2}\left({\mu }_{{\alpha }_{i}}+2\,\log {\sigma }_{{\alpha }_{i}}+1+\log \,2\right)\right),\end{array}$$

30 $$\begin{array}{l}{D}_{{\rm{KL}}}({q}_{\varphi }(\beta )| | p(\beta ))=\mathop{\sum }\limits_{i=1}^{M+D}\left(\exp \left(\frac{1}{2}{\sigma }_{{\beta }_{i}}^{2}-{\mu }_{{\beta }_{i}}\right)\right.\\ \,\,\,\,\left.-\,\frac{1}{2}\left(-{\mu }_{{\beta }_{i}}+2\,\log {\sigma }_{{\beta }_{i}}+1+\log \,2\right)\right),\end{array}$$

31 $${D}_{{\rm{KL}}}({q}_{\varphi }(\widetilde{\theta })| | p(\widetilde{\theta }))=-\,\frac{1}{2}\mathop{\sum }\limits_{i=1}^{M+D}\left(1+2\,\log {\sigma }_{{\widetilde{\theta }}_{i}}-{\mu }_{\widetilde{\theta }}^{2}-{\sigma }_{{\widetilde{\theta }}_{i}}^{2}\right).$$

### Linear measurement function

In the case of a linear observation function with Gaussian noise, the expected log likelihood of the measurements can be written in closed form. Specifically, when ${y}_{{t}_{i}}=Gx({t}_{i})+\eta$, in which $G\in {{\mathbb{R}}}^{d\times D}$ and ${\epsilon } \sim {\mathcal{N}}(0,R)$, we can write

32 $$\begin{array}{c}{{\mathbb{E}}}_{{\mathcal{N}}(m({t}_{i}),S({t}_{i}))}[\log p(\,{y}_{{t}_{i}}|{x}_{{t}_{i}})]=-\frac{1}{2}{({y}_{i}-Gm({t}_{i}))}^{T}{R}^{-1}(\,{y}_{i}-Gm({t}_{i}))\\ \,\,-\frac{1}{2}{\rm{t}}{\rm{r}}({G}^{T}{R}^{-1}GS({t}_{i}))-\,\frac{1}{2}\log |R|-\frac{d}{2}\log 2{\rm{\pi }}.\end{array}$$

This is a useful, well-known result that reduces the variance of gradients of the approximation to the ELBO in equations (17) and (18).

### List of governing equations used in numerical studies

Most of the problems used for benchmarking studies have been used previously to benchmark the performance of methods for governing-equation discovery^27,34^. We list the governing equations, initial conditions, time intervals and the distribution over the probabilistic corruption coefficients. As a reminder, we add the following probabilistic corruption to the dynamics of the first state in the second set of numerical studies: ${\dot{x}}_{1}\leftarrow {\dot{x}}_{1}-\alpha {x}_{2}+\beta$.

Damped linear oscillator:

33 $$\begin{array}{c}\dot{x}=\left[\begin{array}{cc}-0.1 & 2\\ -2 & -0.1\end{array}\right]x,\quad x(0)=\left[\begin{array}{c}2.5\\ -5\end{array}\right],\quad t\in [0,20]\\ \alpha ,\beta  \sim {\mathcal{U}}(-0.25,0.25)\end{array}$$

Damped cubic oscillator:

34 $$\begin{array}{l}\dot{x}=\left[\begin{array}{cc}-0.1 & 2\\ -2 & -0.1\end{array}\right]{x}^{3},\quad x(0)=\left[\begin{array}{c}0\\ -1\end{array}\right],\quad t\in [0,25]\\ \alpha ,\beta  \sim {\mathcal{U}}(-0.15,0.15)\end{array}$$

Coupled linear system:

35 $$\begin{array}{l}\begin{array}{l}{\ddot{x}}_{1}=-\,(4+2){x}_{1}+2{x}_{2}\\ {\ddot{x}}_{2}=2{x}_{1}-(2+4){x}_{2}\end{array},\quad x(0)=\left[\begin{array}{c}0\\ 1\end{array}\right],\\ \dot{x}(0)=\left[\begin{array}{c}0\\ 0\end{array}\right],\quad t\in [0,20]\\ \alpha ,\beta  \sim {\mathcal{U}}(-0.25,0.25)\end{array}$$

Duffing oscillator:

36 $$\begin{array}{l}\begin{array}{l}{\dot{x}}_{1}={x}_{2}\\ {\dot{x}}_{2}=-\,({x}_{1}^{3}-{x}_{1})-0.35{x}_{2}\end{array}\quad x(0)=\left[\begin{array}{c}3\\ 2\end{array}\right],\quad t\in [0,20]\\ \alpha ,\beta  \sim {\mathcal{U}}(-0.25,0.25)\end{array}$$

Selkov glycolysis^57^:

37 $$\begin{array}{l}\begin{array}{l}{\dot{x}}_{1}=-{x}_{1}+0.08{x}_{2}+{x}_{1}^{2}{x}_{2}\\ {\dot{x}}_{2}=0.6-0.08{x}_{2}-{x}_{1}^{2}{x}_{2}\end{array}\quad x(0)=\left[\begin{array}{c}0.7\\ 1.25\end{array}\right],\quad t\in [0,30]\\ \alpha ,\beta  \sim {\mathcal{U}}(-0.04,0.04)\end{array}$$

Lorenz ’63:

38 $$\begin{array}{l}\begin{array}{l}{\dot{x}}_{1}=10({x}_{2}-{x}_{1})\\ {\dot{x}}_{2}={x}_{1}(28-{x}_{3})-{x}_{2}\\ {\dot{x}}_{3}={x}_{1}{x}_{2}-8/3{x}_{3}\end{array}\quad x(0)=\left[\begin{array}{c}-8\\ 7\\ 27\end{array}\right],\quad t\in [0,10]\\ \alpha ,\beta  \sim {\mathcal{U}}(-2.5,2.5)\end{array}$$

Hopf bifurcation:

39 $$\begin{array}{l}\begin{array}{l}{\dot{x}}_{1}=0.5{x}_{1}+{x}_{2}-{x}_{1}({x}_{1}^{2}+{x}_{2}^{2})\\ {\dot{x}}_{2}=-{x}_{1}+0.5{x}_{2}-{x}_{2}({x}_{1}^{2}+{x}_{2}^{2})\end{array}\quad x(0)=\left[\begin{array}{c}2\\ 2\end{array}\right],\quad t\in [0,20]\\ \alpha ,\beta  \sim {\mathcal{U}}(-0.125,0.125)\end{array}$$

### Detailed setup for example 1

This section provides a detailed breakdown of the comparison activities related to state estimation with a known motion model introduced in the first set of numerical studies. To reiterate, in this set of benchmarks, we compared our method (SVISE) to the PF^42^ using an implementation provided by DAPPER^43^ when the form of the motion model is known exactly. This serves as the best-case scenario for standard state-estimation methods.

We use the dynamical system definitions described previously as well as the identity observation function. For each dynamical system, we generate 20 independent datasets and task the algorithms with estimating the mean and variance of the state at each timestamp. Each dataset consists of 128 evenly spaced data points generated by simulating the system forward from the initial condition listed previously using Euler–Maruyama integration assuming a diffusion matrix whose diagonal values are given by 1% of the range of the system and additive zero-centred Gaussian observation noise with a standard deviation that is 10% of the range of the system. We define the range of the system as $1/2\left(\max \left({\{x({t}_{i})\}}_{i=1}^{N}\right)-\min \left({\{x({t}_{i})\}}_{i=1}^{N}\right)\right)$, in which *x*(*t*_*i*_) is the true system state at time *t*_*i*_ assuming zero diffusion.

To assess the performance of the methods, we used the normalized root mean squared error (NRMSE) between the mean estimate for the state and the true state position, ${\rm{NRMSE}}={\left({\sum }_{i=1}^{N}| | x({t}_{i})-{x}_{i}| {| }_{2}^{2}/{\sum }_{i=1}^{N}| | x({t}_{i})| {| }_{2}^{2}\right)}^{1/2}$, in which *x*_*i*_ is the predicted mean of the state at time *i* and *x*(*t*_*i*_) is the true system state.

The state estimators in the DAPPER implementation all require an initial condition. To not unfairly advantage our method, we provided the algorithms we compared ourselves to with the true initial condition for all systems except the Hopf bifurcation (for which we provided the true system state at the time of the first measurement to avoid particles diverging to infinity). Also, we set the initial variance to be 0, as we were providing the PF with the exact initial condition. For the Hopf bifurcation, so as to not unfairly advantage our method, we only measured performance for estimates of the state provided after 10 s (thereby giving the PF sufficient burn-in time).

For all comparisons in this section, we used the default settings provided by DAPPER v1.3.0 (ref. ^43^), with the exception of choosing 1,000 particles. The results were summarized in the main text and in Fig. 2a.

### Detailed setup for example 2

This section provides a detailed description of the comparison activities related to state estimation with modelling errors that was introduced in the second set of numerical studies. To reiterate, in contrast to the comparisons described in the previous section, in this set of comparisons, we introduce a corruption to the differential equations that the PF was not made aware of. If the uncorrupted dynamics are given by $\dot{x}\in {{\mathbb{R}}}^{D}$, in which ${\dot{x}}_{i}$ is the differential equation governing the dynamics of the *i*th component of *x*, we corrupt the dynamics of the first state by setting ${\dot{x}}_{1}\leftarrow {\dot{x}}_{1}-\alpha {x}_{2}+\beta$ while all other states are left uncorrupted. The corruption terms *α* and *β* are sampled from zero-centred uniform distributions using the bounds listed previously. In the case of the cubic oscillator, for example, this introduces a small amount of extra linear damping to the system. We use the same settings as in the previous benchmarks except for the fact that we generate data by simulating with the corrupted dynamics. For the PF, we set reg = 2.4 and NER = 0.3. These settings were found by starting with the DAPPER defaults and manually tuning.

Looking to Fig. 2b, we see that our method outperforms the PF on average at this level of corruption. To reiterate what was discussed in the main text, we believe that these results make clear the usefulness of this work. Even in the presence of mild modelling error, our method outperforms standard state-estimation tools. As modelling error is an unavoidable reality in many systems of interest, our method for state estimation has the potential to be useful.

### Detailed setup for example 3

To generate data, we used an immersed boundary projection method codebase for solving the two-dimensional incompressible Navier–Stokes equations^58^. We generated 1,953 evenly spaced snapshots each with dimension of 596,602 over the time interval [61, 256.2]. We assume a Gaussian likelihood with a constant variance of 10^−3^. In this problem, 38 POD modes were required to capture 90% of the variance. The fully connected neural network model for the drift was chosen to have one hidden layer with 128 hidden units and tanh nonlinearities. We used a batch size of 64, set *K* = 100, *M* = 100 and used 500 basis functions to approximate the posterior of the state. To reiterate what was mentioned previously, looking to Fig. 3, we see that we are successfully able to infer a probabilistic ROM for the flow.

### Example 4: symbolic-governing-equation discovery benchmarking

In this section, we compare our method to standard governing-equation learning algorithms from the literature on the suite of benchmark systems listed previously in Methods. For all experiments in this section, we require *g* to be the identity measurement function so that methods from the literature can be applied. Also, we provide our method with no knowledge of the underlying dynamics (that is, we set ${f}_{\varnothing }\,=\,0$ and ${\Sigma }_{\varnothing }=0$). We compared our method to the algorithm for sparse identification of nonlinear dynamics (SINDy), SINDy with sequentially thresholded least squares regression (SINDy-STLSQ)^27^, SINDy with sparse relaxed regularized regression (SINDy-SR3)^59^ and Ensemble-SINDy (ENS-SINDy)^60^ using implementations provided by PySINDy^61,62^. Each method is given a dictionary of functions containing all polynomials up to order 5.

For each system, we generate 40 independent datasets and task the algorithms with recovering the underlying governing equations. These 40 independent datasets are split up equally into two cases: (1) the low-noise, low-data regime and (2) the high-noise, large-data regime. In the low-noise, low-data regime, we generate 16 data points for the damped linear and damped cubic oscillators, 32 data points for the Hopf bifurcation, Selkov glycolysis model and Duffing oscillator, and 64 data points for the Lorenz ’63 and coupled linear oscillator. Each data point is sampled by assuming zero-centred Gaussian noise with a standard deviation that is 1% of the range of the system. In the high-noise, large-data regime, we generate 2,048 evenly spaced data points for each dataset assuming zero-centred Gaussian noise with a standard deviation that is 25% of the range of the system. We again define the range of the system as $1/2\left(\max \left({\{x({t}_{i})\}}_{i=1}^{N}\right)-\min \left({\{x({t}_{i})\}}_{i=1}^{N}\right)\right)$, in which *x*(*t*_*i*_) is the true system state at time *t*_*i*_.

The reconstruction error and the number of incorrect terms are metrics commonly used in compressive sensing to validate the efficacy of sparse learning algorithms; see, for example, refs. ^63,64^. The reconstruction error is defined as ${\rm{RER}}=| | \theta -\widehat{\theta }| {| }_{2}/| | \theta | {| }_{2}$, in which *θ* is the true vector of sparse weights that we are trying to estimate and $\widehat{\theta }$ is our estimate for the set of sparse weights. For all estimates of the reconstruction error, we use the mean of the estimated posterior for the weights. The reason for looking at both metrics is that some algorithms may achieve a low reconstruction error without correctly pruning weights.

**Extended Data Fig. 1:**
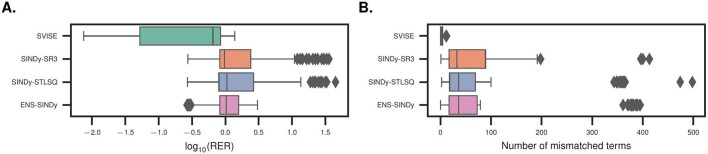
Identifying missing terms. **a**, Reconstruction error. **b**, Number of mismatched terms. Our method (SVISE) outperforms state-of-the-art algorithms for governing-equation discovery in the high-noise and low-data regimes.

Summary plots for benchmarking in the high-data regime are provided in Extended Data Fig. 1. Detailed benchmarking tables broken down by each system can be found in Extended Data Tables 2–5. In these tables, the error bars for all terms are given by one standard deviation from the mean. A dash indicates that the equation-recovery algorithm predicted that the governing equations were given by $\dot{x}=0$.

It can be seen from the results that, as well as improving on reconstruction error, often by more than an order of magnitude, our method was able to identify the correct functional form of the governing equations far more frequently than the methods with which we compared in both the low-data and high-noise regimes. These results are notable because, in many problems for which governing equations are not available, we only have access to noisy/limited data.

Looking more closely at Extended Data Table 2, we see that the proposed method (SVISE) outperformed all other methods in terms of reconstruction error in the low-data regime except on the Duffing oscillator problem, for which our approach was outperformed by ENS-SINDy. For this benchmark, our approach still outperformed ENS-SINDy in terms of the number of mismatched terms. Looking now to Extended Data Table 3, we see that the proposed method outperformed all other methods in terms of the number of mismatched terms for all but the Lorenz ’63 benchmark; however, the SINDy-STLSQ method achieved this lower number of incorrect terms score by often ignoring the dynamics in the third state.

In the high-noise regime, we see that SVISE outperformed the methods we compared with in terms of RER on all but the Duffing oscillator benchmark, for which our approach was again outperformed by ENS-SINDy (see Extended Data Table 4). For this benchmark, our method was again the clear winner in terms of the number of mismatched terms (see Extended Data Table 5).

Taken together, these results demonstrate that the method introduced in this work is a good choice for governing-equation discovery in the low-data and high-noise regimes. Also, although the methods we compared ourselves to in this section require that the measurement function is identity (that is, the full state vector is measured), our method is applicable to cases with arbitrary observation functions.

### Example 5: high-dimensional, spatially extended differential-equation discovery with low-rank observation matrices

We now consider the problem of recovering the underlying governing equations for the Lorenz ’96 system with 1,024 states using a low-rank observation matrix. The Lorenz ’96 model is a set of coupled, chaotic, ODEs designed to be a simplified model of the climate along a line of constant latitude^65^. The governing equations for this system are given by ${\dot{x}}_{k}={x}_{k-1}({x}_{k+1}-{x}_{k-2})-{x}_{k}+10$, for which the boundary conditions are assumed to be periodic (*k* = 1, 2,…, 1,024).

**Extended Data Fig. 2:**
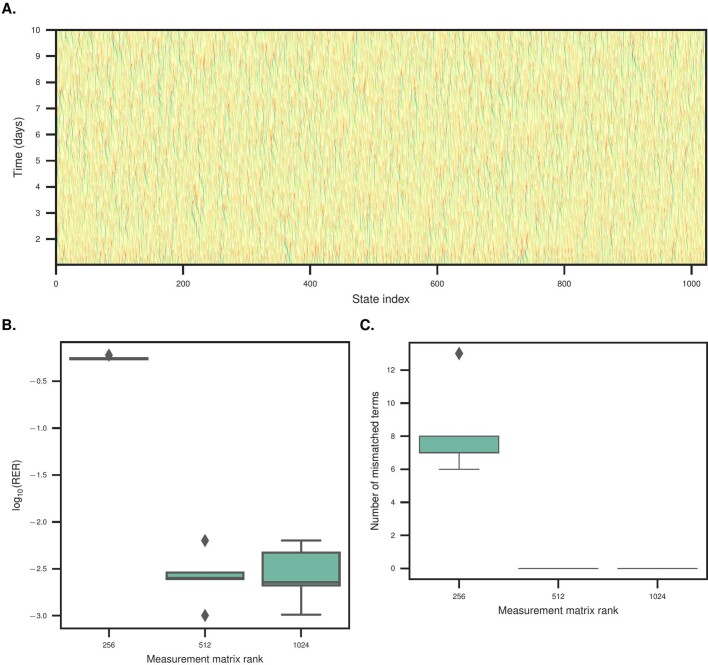
Experiment on discovering governing equations for a high-dimensional system with a low-rank observation matrix. **a**, Visualization of Lorenz ’96 full underlying state data with 1,024 states. **b**, Reconstruction error for varying observation matrix ranks. **c**, Number of mismatched terms for varying observation matrix ranks. In this experiment, we assumed that the governing equations were a sparse linear combination of basis functions from the dictionary of quadratic polynomial functions of the node state and its four closest neighbours to the left and right of the node. We were able to successfully recover the exact functional form of the Lorenz ’96 governing equations using an observation matrix whose rank was half that of the full underlying state.

We generated observation matrices of rank *r* using the expression $g(x)=\left({r}^{-1}{\sum }_{i=1}^{r}{u}_{i}{u}_{i}^{T}\right)x$, in which each ${u}_{i}\in {{\mathbb{R}}}^{D}$ is a random vector sampled from a standard normal distribution. We studied the performance of our approach in which the rank of the observation matrix is 256, 512 and 1,024. For each experiment, we used 512 snapshots over the time interval of 0 to 10 corrupted by noise that is 2% of the range of the system. We make the assumption that the dynamics are given by ${\dot{x}}_{i}={{\mathcal{P}}}_{2}({x}_{i-2},{x}_{i-1},{x}_{i},{x}_{i+1},{x}_{i+2})\theta$, in which ${{\mathcal{P}}}_{2}:{{\mathbb{R}}}^{5}\to {{\mathbb{R}}}^{1\times M}$ returns all quadratic polynomial functions that are a function of *x*_*i*_ and its two closest neighbours to the left and right of the node. Although we found that we were able to exactly recover the underlying functional form of the governing equations with an observation matrix whose rank was half the dimensionality of the state, further work is required to theoretically establish conditions under which the governing equations can be exactly recovered. These results are summarized in Extended Data Fig. 2.

Because many real-world systems for which governing equations are challenging to derive from first principles are both (1) high-dimensional and (2) challenging to measure, we believe this to be a useful result. Our method has a computational cost that scales linearly in the state dimension and can be applied given an arbitrary observation function. This result opens the door for equation discovery in systems that were previously believed to be too large and/or difficult to measure.

### Example 6: governing-equation discovery for second-order systems with unobserved states

We now examine the problem of learning governing equations of second-order systems using only displacement/position measurements. For many physical systems, we often only have access to position measurements (that is, through GPS/Vicon camera measurements) for certain states and/or accelerometer measurements for other states. To apply existing methods from the literature to this class of problem, it becomes necessary to estimate velocities and accelerations using finite-difference approximations^66^ in the preprocessing stage or use the weak form of the governing equations^67^. The proposed method can be directly applied to such problems without resorting to finite differences or the weak form.

We consider the task of learning the governing equations of the coupled linear oscillator defined in governing-equations definitions discussed previously. We generate 32 measurements of the system position corrupted by 10% measurement noise. Assuming that the system is autonomous and second order, we know that the dynamics must be governed by second-order differential equations, which are functions of both the position and the velocity.

**Extended Data Fig. 3:**
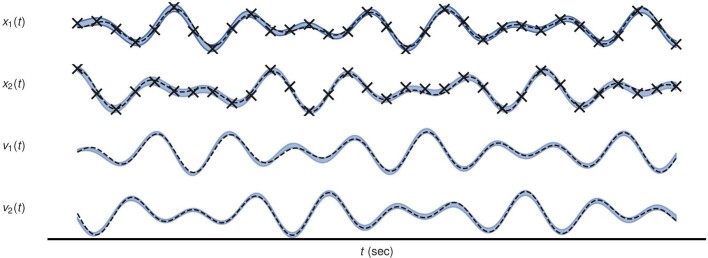
Governing-equation discovery for second-order system using only position measurements. The dashed lines indicate the true oscillator state and the shaded blue regions indicate two standard deviations from the mean of the underlying state estimate. The state positions are marked by *x*_1_ and *x*_2_ and the state velocities are marked by *v*_1_ and *v*_2_. Here only 32 measurements of the oscillator position corrupted by noise that was 10% of the range of the system state were available.

Using a dictionary containing polynomials up to order five in the state and velocity, we were able to infer both the structural form of the underlying governing equations as well as estimates for the uncertainty in the parameters. The governing equations inferred by our method are provided below (only the mean coefficients are shown):

40 $$\begin{array}{l}{\ddot{x}}_{1}=-\,5.23{x}_{1}+1.74{x}_{2},\\ {\ddot{x}}_{2}=1.61{x}_{1}-5.45{x}_{2},\end{array}$$

which is relatively close to the true functional form of the underlying governing equations. Extended Data Fig. 3 shows the measurements and underlying state inferred by our method. It can be seen that the probabilistic state estimate agrees well with the true trajectory.

This experiment demonstrates the use of our method in practical situations in which we only have access to partial measurements of the state for second-order systems. So far, we have only considered problems in which the observation function is linear. In the next section, we consider a problem with a nonlinear observation function in which there are fewer observations than states.

### Example 7: binary black hole system from gravitational-wave measurements

The binary-black-hole modelling problem is concerned with estimating black-hole orbital trajectories using observations of gravitational waves^68^. In comparison with the examples considered so far, this problem involves a nonlinear observation likelihood and the number of observations is less than the number of states. Moreover, in this problem, it is not clear what choice of basis functions might be appropriate for approximating the underlying dynamics. We shall demonstrate that it is indeed possible to use a neural network in place of a linear combination of basis functions, as was mentioned when outlining our approach.

Traditional approaches to solving this problem typically involve reconciling gravitational-wave measurements with the complex partial differential equations that govern their dynamics. This is typically a computationally expensive undertaking. In this section, we attempt to infer the governing equations that were likely to have generated the waveform observations simultaneously to the underlying orbital trajectories. We consider a special case of the binary-black-hole modelling problem for an extreme-mass-ratio system (that is, in which the mass of one object is far greater than the other); see Keith et al.^69^ and references therein for more details on this problem.

Centring the origin of the coordinate system at the more massive object, the dynamics can be written as a set of coupled differential equations in terms of the angle of the smaller object with respect to the *x* axis, *δ*(*t*), and the anomaly, *χ*(*t*),

41 $$\dot{\delta }=\frac{(p-2-2e\cos \chi ){(1+e\cos \chi )}^{2}}{{p}^{3/2}{\left({(p-2)}^{2}-4{e}^{2}\right)}^{1/2}},$$

42 $$\dot{\chi }=\frac{(p-2-2e\cos \chi ){(1+e\cos \chi )}^{2}{(p-6-2e\cos \chi )}^{1/2}}{{p}^{2}{\left({(p-2)}^{2}-4{e}^{2}\right)}^{1/2}},$$

in which *e* = 0.5 is the eccentricity and *p* = 100 is the semilatus rectum. The angle and anomaly relate to the orbital trajectory according to

43 $$\left[\begin{array}{c}x(t)\\ y(t)\end{array}\right]=\frac{p}{(1+e\cos \chi (t))}\left[\begin{array}{c}\cos \delta (t)\\ \sin \delta (t)\end{array}\right].$$

As mentioned previously, in practice, we cannot observe the state variables directly. Instead, we only have access to noisy gravitational-waveform measurements, *w*(*t*),

44 $$w(t)=\sqrt{\frac{4\pi }{5}}\left(\frac{{{\rm{d}}}^{2}}{{\rm{d}}{t}^{2}}x{(t)}^{2}-\frac{{{\rm{d}}}^{2}}{{\rm{d}}{t}^{2}}y{(t)}^{2}\right).$$

Note that we consider only the dominant (2,2)-mode gravitational waveforms.

Given gravitational-waveform observations, *w*(*t*), our goal is to reconstruct the underlying orbital trajectories, *x*(*t*) and *y*(*t*), and to infer with an approximate forward model that can be used to forecast future orbital trajectories. Rather than working directly in the trajectory coordinates, we will infer a SDE in terms of the orbital parameters, *δ*(*t*) and *χ*(*t*), in which *χ*(*t*) is the anomaly and *δ*(*t*) is the angle with respect to the *x* axis of the smaller object. Following the parametrization suggested by Keith et al.^69^, we model the drift as

45 $${f}_{\theta }(t,\delta ,\chi )=\frac{{(1+e\cos \chi )}^{2}}{M{p}^{3/2}}(1+{{\mathcal{F}}}_{\theta }(\cos \chi )),$$

in which ${{\mathcal{F}}}_{\theta }$ is a fully connected neural network with two outputs, *M* is the mass of the more massive object, *e* = 0.5 is the eccentricity and *p* = 100 is the semilatus rectum. We use two hidden layers, each with 128 hidden units and tanh nonlinearities. We collect 1,000 evenly spaced gravitational-waveform observations, *w*(*t*), over the interval [0, 0.6 × 10^5^] corrupted by Gaussian noise with a standard deviation of 10^−3^. We also provide our algorithm with the initial condition of the underlying state at the first observation time. We place a sparsity-inducing prior on the diagonal of the diffusion term.

**Extended Data Fig. 4:**
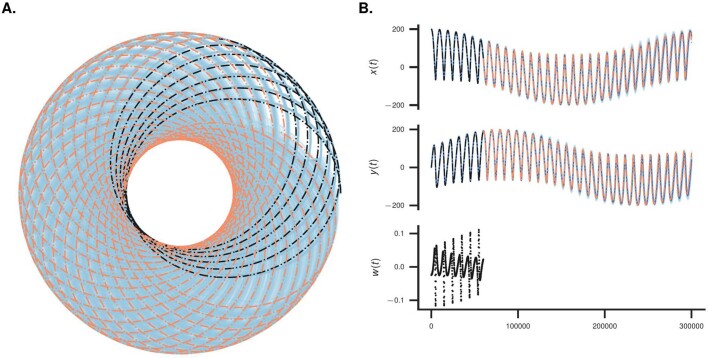
Binary-black-hole modelling problem. **a**, Orbital trajectory estimates. **b**, Trajectory estimates versus time. In this experiment, we approximate the dynamics using a neural network. This is a useful model in situations in which it is not clear what dictionary of basis functions is appropriate. The left figure shows the predicted orbital trajectories in the plane of the orbit and the right figure shows the predicted orbital trajectories as a function of time. The black lines indicate the data windows, the orange lines indicate the testing data and the blue intervals indicate probabilistic predictions from our model. Note that we only have access to the waveform observations, *w*(*t*), up to time 0.6 × 10^5^ in training. We are able to accurately estimate the states and provide probabilistic estimates for the orbital trajectories well into the future, despite not having access to the underlying governing equations.

We choose a batch size of 256, a learning rate of 10^−2^, 20 samples from the variational posterior, 1,000 warm-up iterations, 100 basis functions to approximate the posterior over the state and decayed the learning rate by 0.9 every 500 iterations; see Methods for more details. The results are summarized in Extended Data Fig. 4. We find that we are able to infer a reasonable model for the orbital trajectory while estimating the state. We reiterate that, in contrast to previous approaches to solving this problem, we were not required to solve any differential equations in training. Also, our approach provides probabilistic predictions for the orbital trajectories.

Like for the Lorenz ’96 example, further work is required to determine when uncovering the governing equations is possible for general nonlinear observation functions, particularly when there are fewer observations than states. For example, in this problem, we found that the success of our approach relied heavily on the carefully designed parametrization for the drift term suggested by Keith et al.^69^. It is also worth mentioning that, because we are estimating the parameters of the neural network using maximum-likelihood estimation, we expect to systematically underestimate uncertainty. Future work could consider placing priors on the neural-network parameters and performing approximate variational inference over said parameters to more accurately capture uncertainty.

## Online content

Any methods, additional references, Nature Portfolio reporting summaries, source data, extended data, supplementary information, acknowledgements, peer review information; details of author contributions and competing interests; and statements of data and code availability are available at 10.1038/s41586-023-06574-8.

## Figures and Tables

**Extended Data Table 1:**
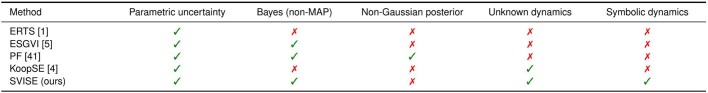
Assumption comparison table

Capability comparison between the extended Rauch–Tung–Striebel smoother (ERTS), the exactly sparse Gaussian variational inference method for state estimation (ESGVI), the PF and our approach, SVISE. We note that ours is the only method that allows for symbolic-governing-equation discovery as well as a Bayes (non-MAP) state estimate.

**Extended Data Table 2:**
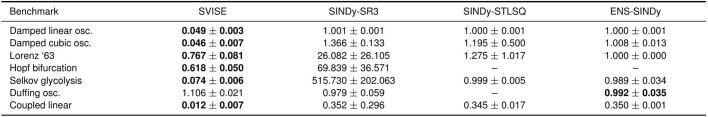
RER in the low-data regime

Reconstruction error averaged over 20 independent trials. Error bars given by 1 standard deviation.

**Extended Data Table 3:**
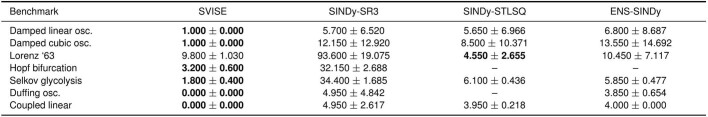
Number of mismatched terms in the low-data regime

Number of mismatched terms averaged over 20 independent trials. Error bars given by 1 standard deviation.

**Extended Data Table 4:**
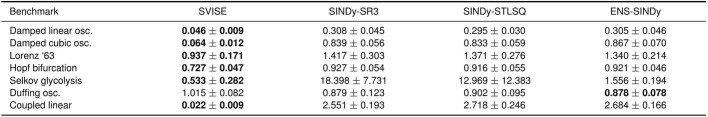
RER in the high-noise regime

Reconstruction error averaged over 20 independent trials. Error bars given by 1 standard deviation.

**Extended Data Table 5:**
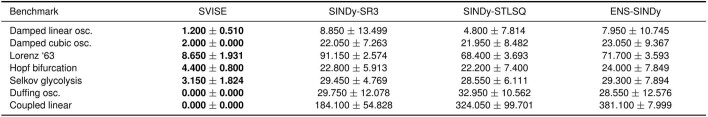
Number of mismatched terms in the high-noise regime

Number of mismatched terms averaged over 20 independent trials. Error bars given by 1 standard deviation.

## Data Availability

The data in the paper and the Supplementary Information are available at https://github.com/coursekevin/svise.

## Code availability

The software library that was developed to generate the results along with documentation is available at https://github.com/coursekevin/svise.
